# Extended Follow-Up Outcomes from Pooled Prospective Studies Evaluating Efficacy of Interstitial Alpha Radionuclide Treatment for Skin and Head and Neck Cancers

**DOI:** 10.3390/cancers16132312

**Published:** 2024-06-24

**Authors:** Aron Popovtzer, Aviram Mizrachi, Mark A. D’Andrea, Noam A. VanderWalde, Noga Kurman, Eli Rosenfeld, Ran Ben-Hur, Salvatore Roberto Bellia, Giacomo Feliciani, David Silvern, Anna Sarnelli, Matthew T. Ballo, Pradeep Patra, Gil’ad N. Cohen, Antonio L. Damato, Yotam Shkedy, Robert B. Den, Christopher A. Barker, Tomer Charas, Nir Hirshoren

**Affiliations:** 1 Hadassah Medical Center, Ein Kerem 9112001, Israel; 2 Rabin Medical Center, Derech Ze’ev Jabotinsky St., 39, Petah Tikva 4941492, Israel; 3 University Cancer Centers, 12811 Beamer Rd, Houston, TX 77089, USA; 4 West Cancer Center, 7945 Wolf River Boulevard, Germantown, TN 38138, USA; 5 IRCCS Istituto Romagnolo per lo Studio dei Tumori (IRST) “Dino Amadori”, 47014 Meldola, Italy; 6 Memorial Sloan Kettering Cancer Center, 1101 Hempstead Turnpike, Uniondale, NY 11553, USA; gil’; 7 Rambam Healthcare Campus, HaAliya HaShniya St 8, Haifa 3109601, Israel; 8 Department of Radiation Oncology, Jefferson University, 1025 Walnut Street, Philadelphia, PA 19107, USA; 9 AlphaTau Medical, Kiryat HaMada St 5, Jerusalem 9777605, Israel

**Keywords:** alpha particles, cutaneous squamous cell carcinoma, basal cell carcinoma, skin cancer, head and neck cancer, alpha-emitter radiation therapy, DaRT

## Abstract

**Simple Summary:**

While the initial favorable efficacy and safety profile for a novel diffusing alpha-emitter radiation therapy (Alpha DaRT) have been previously demonstrated, the longer-term safety and durability of the treatment are unknown. In this pooled analysis of 81 treated head and neck or skin tumors from four clinical trials with a median follow-up of 14 months, a complete response was observed in 89% of treated lesions, two-year local recurrence-free survival was 77%, and there were no grade 2 or higher late toxicities observed. These results suggest that Alpha DaRT is a safe and effective treatment for skin and head and neck tumors.

**Abstract:**

The initial favorable efficacy and safety profile for Alpha DaRT have been demonstrated (NCT04377360); however, the longer-term safety and durability of the treatment are unknown. This pooled analysis of four prospective trials evaluated the long-term safety and efficacy of Alpha DaRT for the treatment of head and neck or skin tumors. A total of 81 lesions in 71 patients were treated across six international institutions, with a median follow-up of 14.1 months (range: 2–51 months). Alpha DaRT sources were delivered via a percutaneous interstitial technique and placed to irradiate the tumor volume with the margin. The sources were removed two to three weeks following implantation. A complete response was observed in 89% of treated lesions (n = 72) and a partial response in 10% (n = 8). The two-year actuarial local recurrence-free survival was 77% [95% CI 63–87]. Variables, including recurrent versus non-recurrent lesions, baseline tumor size, or histology, did not impact long-term outcomes. Twenty-seven percent of patients developed related acute grade 2 or higher toxicities, which resolved with conservative measures. No grade 2 or higher late toxicities were observed. These data support the favorable safety profile of Alpha DaRT, which is currently being explored in a pivotal US trial.

## 1. Introduction

The radiotherapeutic management of recurrent skin and head and neck cancers often poses a challenge for the clinician, where the aim is to optimize tumor control by delivering tumoricidal doses while at the same time minimizing treatment-related morbidity, especially in the setting of prior therapy such as surgery and/or radiation therapy. For patients with cutaneous cancer presenting with high-risk features as defined by the NCCN [1], the risk of local recurrence can be as high as 53%. Mortality from cutaneous and recurrent head and neck cancer is often related to uncontrolled regional disease, and metastatic disease has been associated with mortality rates of greater than 70% [2,3,4]. 

Irradiation of skin tumors that previously underwent multiple prior excisions with or without radiotherapy can also compromise the integrity of the skin, leading to severe scarring, poor wound healing, and suboptimal cosmesis [5].

Diffusing alpha-emitting Radiation Therapy (DaRT) is a novel method of delivering alpha radiation to solid tumors using the intratumoral placement of wires impregnated with radium-224 sources (3.7-day half-life). The decay of the primary isotope starts a decay chain of alpha-emitters, resulting in tumor cell death. The mechanism of action has been detailed in preclinical studies [6,7,8,9]. DaRT combines the advantages of local intra-tumoral irradiation with the destructive power of alpha particles, which is recognized to be significantly more potent than other forms of radiation. Additionally, due to the short range of alpha particles in tissue, most of the radiation absorption occurs within the tumor, and the surrounding healthy tissue is preferentially spared. 

We have previously reported preliminary outcomes of diffusing alpha-emitter radiation therapy (Alpha DaRT) for patients with recurrent skin and selected head and neck tumors, where we demonstrated excellent initial tumor responses with low rates of severe acute toxicity in individual studies [10,11]. In this report, we pooled patients from multiple international prospective studies who were treated with Alpha DaRT and were subsequently followed for up to four years after treatment. Therefore, we now report the positive tumor control and toxicity outcomes that continued to be demonstrated even with longer-term follow-up.

## 2. Materials and Methods

Data from four prospective studies designed to assess the efficacy, feasibility, and safety of Alpha DaRT administration as a monotherapy for the treatment of malignant skin and superficial soft tissue tumors (ClinicalTrials.gov identifiers: NCT03015883, NCT04534127, NCT04377360, and NCT03889899) were collected and compiled into one database. 

Eligibility for protocol enrollments and exclusion criteria have been described in prior publications [10,11]. The administration of Alpha DaRT and planning workflow have also been described in detail [12]. In brief, eligible patients had biopsy-proven recurrent or unresectable squamous cell or basal cell carcinoma of the skin or squamous cancer of the head and neck, who either previously failed or were medically unfit for definitive therapy, or who had declined the standard of care therapy. These eligible patients underwent a volumetric assessment of the tumor via a computed tomographic (CT) radiation therapy planning scan. The volumetric images were used to generate the plan to deliver Alpha DaRT by defining the optimal number, size, and location for Alpha DaRT source placement to irradiate the tumor volume with a margin. Using these planning parameters, the Alpha DaRT sources were inserted by surgeons or radiation oncologists. Immediately after placement, a standard radiation therapy planning CT was performed to assess source positions within the tumor. Coverage was evaluated using a technique involving placing virtual sources on top of the actual sources seen on the scan. Each virtual source is programmed to provide its dose in the 3D volumetric space. This allows the calculation of a dose volume histogram (DVH) of a given contour (i.e., tumor, organ at risk). If a cold spot was found (a voxel on the scan within the contour receiving less than the prescription dose), additional sources were inserted to ensure the whole tumor was irradiated. A physical dose of 10 Gy was prescribed [13,14].

Two to three weeks after the placement of the Alpha DaRT sources, the sources were removed. Tumor response to Alpha DaRT was assessed for response by RECIST V1.1, with only the irradiated tumor considered a target lesion. The assessment was performed approximately 10–12 weeks after the removal of the device using physical inspection and CT imaging.

For this report, several endpoints were evaluated, including two-year toxicity outcomes, the local control rate, and the overall survival rate (OS). The local control rate was defined as the time from response—complete response (CR), partial response (PR), or stable disease (SD)—to recurrence. The overall survival was defined as the time from the first (in cases where more than one lesion was treated) Alpha DaRT insertion to the time of death for any reason. 

Statistical analyses were performed using SAS^®^ V9.4 (SAS Institute, Cary, NC, USA). The analyses of the study endpoints were descriptive in nature. All statistical tests performed were two-sided. The required significance level of the findings was equal to or lower than 5%. Where confidence limits are appropriate, the 95% confidence level is reported. Nominal *p*-values and unadjusted confidence intervals (CI) are also presented. Demographic and baseline variables are tabulated. Continuous variables are summarized by a mean, standard deviation, minimum, median, and maximum, whereas categorical variables are summarized by a count and percentage. Because more than one tumor was treated for several patients, some parameters, such as demographic data, are presented on a patient basis, while other parameters, such as tumor characteristics, are presented on a lesion basis.

The overall survival outcomes were calculated using Kaplan–Meier analysis. Patients still alive were considered right-censored, and the last known date the patient was confirmed alive was used in the analyses. Hazard ratios for age, BMI, sex, and histopathology were calculated to assess if these parameters affected the OS.

The time from response to local recurrence was analyzed in the same manner. Patients with responses that were not recurrent at the time of data collection were considered right censored, and the last known date were used in the analyses. Hazard ratios for clustered data for age, BMI, sex, histopathology, recurrence of tumor, and baseline gross tumor volume were calculated to assess if these parameters affected the recurrence rate.

Overall survival outcomes were calculated on a patient basis, while the time from response to recurrence was calculated on a lesion basis.

Toxicity was scored according to the Common Terminology Criteria for Adverse Events (CTCAE v5). Acute toxicity was defined as complications that developed within three months from Alpha DaRT insertion, and late toxicity was defined as complications that presented after this point in time. Tumor control was assessed using RECIST criteria evaluated by serial radiographic imaging or clinical assessments.

## 3. Results

### 3.1. Patient Characteristics

Table 1 presents the number of patients and lesions per study, clinical site, and protocol number. Seventy-one patients, with a total of 81 lesions, are included in this report. Table 2 summarizes the demographic and baseline characteristics of this pooled cohort of patients, and Table 3 summarizes the characteristics of the pooled tumors. A total of 56% of the treated tumors were squamous cell carcinoma (SCC), and the remaining 44% were basal cell carcinoma (BCC). The mean gross tumor volume was 2·2 cm^3^ (SD: 4·38) and ranged between 0·03 cm^3^ and 33·9 cm^3^. The most frequent tumor locations were the nose (27%), the face (16%), the ears (14%), and the scalp (12%).

### 3.2. Toxicity Outcomes

Nineteen (27%) patients developed grade 2 acute treatment-related toxicity, which resolved in almost all patients using conservative measures such as antibiotics or topical steroid applications. One patient had grade 3 toxicity, a case of elevated blood pressure on the day of the procedure. The median time to resolution of the grade 2 or higher toxicities was 19 days. The specific acute skin-related toxicities are summarized in Table 4. There were no late complications observed after three months, further corroborating the long-term safety of Alpha DaRT.

### 3.3. Tumor Control

**Figure 1:**
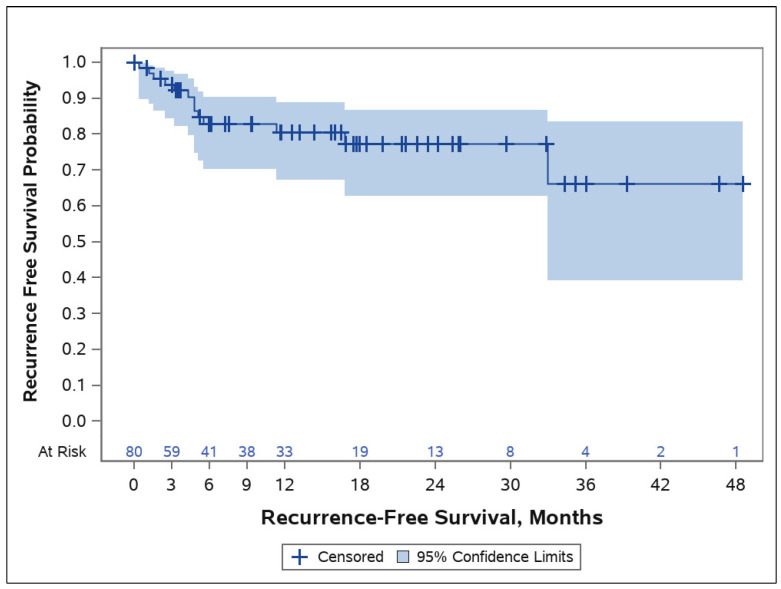
Graph depicting recurrent-free survival in months. The two-year actuarial local recurrence-free survival (LRFS) was 77%.

The median patient follow-up was 14.1 months (range: 2–51 months). A complete response was observed in 89% of treated lesions (n = 72) and a partial response in 10% (n = 8), while one patient was not evaluable. As shown in Figure 1, the two-year actuarial local recurrence-free survival (LRFS) was 77% [95% CI 63–87]. Variables, including recurrent versus non-recurrent lesions, baseline tumor size, or histology, did not impact long-term outcomes. The effect of the tumor location was not formally tested due to the diversity of locations treated. However, recurrences took place across a range of locations, diminishing any suspicion that location impacted outcomes.

## 4. Discussion

We analyzed data pooled from several ongoing prospective studies that are evaluating the use of Alpha DaRT for squamous cell carcinoma or basal cell carcinoma of the skin, head, and neck. We found durable tumor control achieved at the implanted site, with a two-year local recurrence-free survival of 77%. This is higher than the two-year local recurrence-free survival from other radiotherapeutic studies. For example, the two-year local control of external beam radiotherapy for recurrent head and neck cancer is 25% [15], and the two-year local control of high-dose brachytherapy for non-melanoma skin cancers is 53% [16]. These encouraging findings are consistent with expected tumor responses seen with alpha radiation, which possesses greater radiobiologic potency compared to other forms of radiation. Given the high radiobiologic potential for tumor eradication associated with alpha irradiation, such intervention may more effectively address radioresistant tumor clones that are often observed among patients failing radiation using conventional radiotherapy techniques. 

Despite these high dose levels, the findings we report here with extended follow-up confirm our initial short-term observations of a low incidence of complications, including wound healing, soft tissue necrosis, or fibrosis leading to dysfunctional outcomes [10,11]. The lower incidence of long-term soft tissue complications may be related to the very limited diffusion of the alpha particles in the treated tissue, leading to a highly focused delivery dose. This is in contrast to what is expected with external beam radiotherapy, where significantly broader areas of tissues are exposed to a high dose of irradiation. 

Inspired by the promising findings reported in prior Alpha DaRT publications [10,11] and the longer follow-up analyses reported here, there is now an ongoing multicenter trial called “A Clinical Study to Assess the Efficacy and Safety of Alpha DaRT224 for the Treatment of Patients with Recurrent Cutaneous Squamous Cell Carcinoma” (NCT05323253). The co-primary endpoints for this trial are the objective response rate established by the best overall response and the rate of durable response at six months from the initial response. This pivotal trial will help provide a new standard of care for patients with few treatment options.

Outside the context of the patient population noted in this report, Alpha DaRT is being studied for multiple cancers worldwide, including pancreatic, colorectal, prostate, breast, lung, and vulvar cancer. While these are initial feasibility and safety studies, preclinical studies have indicated that Alpha DaRT should be effective across tumor histologies.

In addition, Alpha DaRT has been evaluated in both preclinical [17,18] and clinical studies [19] to augment cancer-specific immune responses and synergize with checkpoint inhibitors and other immune stimulatory agents. Given the rich immunogenic nature of cutaneous squamous cell carcinoma as well as the success of checkpoint inhibitors for the management of this disease, Alpha DaRT is well positioned to be integrated with these agents in future trials. Currently, there is an ongoing trial for metastatic head and neck squamous cell carcinoma investigating combining Alpha DaRT with anti-PD1 therapy (NCT05047094). These combination therapies have the potential to both increase the durability of the immune response as well as increase the number of responding patients. Taken together, this represents an exciting and novel approach to the management of a complex disease. Outside the context of the patient population noted in this report, Alpha DaRT is being studied for multiple cancers worldwide, including pancreatic, colorectal, prostate, breast, lung, and vulvar cancer. While these are initial feasibility and safety studies, preclinical studies have indicated that Alpha DaRT should be effective across tumor histologies. 

## 5. Conclusions

The results of this study demonstrate that Alpha DaRT treatment is safe even beyond the acute period, with no moderate or severe late toxicities observed. Short-term local responses to Alpha DaRT treatment led to longer-term control. Further follow-up and additional clinical studies are ongoing to provide further characterization of the safety profile for this novel cancer therapy. However, the lack of moderate or severe toxicities, together with the longer-lasting responses shown in this cohort, is promising and suggests that larger studies may offer a safe and effective treatment for head and neck and skin cancer patients with limited options.

## Figures and Tables

**Table 1:** Number of Patients and Lesions by Protocol and Clinical Site.

Protocol Number	Clinical Site	No. of Patients	No. of Lesions
CTP-CMN-02	Hadassah Medical Center	28	33
CTP-CMN-03	Rambam Healthcare Campus	10	10
CTP-SCC-00	Rabin Medical Center	18	22
CTP-SCC-00	IRCCS Istituto Romagnolo per lo Studio dei Tumori (IRST) “Dino Amadori”	5	6
CTP-SCC-MSK-00	University Cancer Centers	8	8
CTP-SCC-MSK-00	West Cancer Center	2	2
Total	71	81	

**Table 2:** Patient Baseline Characteristics Summary Table.

Patient Characteristics, Statistics	Results	N
Age (years), mean ± SD (range)	76.3 ± 11.7 (57.8–92.0)	71
Gender, n (%)		71
Female	25 (35%)	
Male	46 (65%)	
BMI, mean ± SD (range)	26.9 ± 5.8 (17.0–42.0)	66

**Table 3:** Tumor Baseline Characteristics Summary Table.

Tumor Characteristic, Statistics	Results	N
Histopathology, n (%)		81
BCC	36 (44%)	
SCC	45 (56%)	
Recurrent tumor, n (%)		80
Recurrent	38 (47%)	
Newly diagnosed	42 (52%)	
Tumor location, n (%)		81
Ear	11 (14%)	
Extremity	8 (10%)	
Eyelid	2 (2%)	
Face	13 (16%)	
Lip	4 (5%)	
Neck	1 (1%)	
Nose	22 (27%)	
Oral cavity	5 (6%)	
Scalp	10 (12%)	
Tongue	4 (5%)	
Torso	1 (1%)	
Duration of disease for recurrent tumors (months), mean ± SD (range)	70.3 ± 69.5 (1.3–285)	38
Baseline Gross Tumor Volume (cm^3^), mean ± SD (range)	2.23 ± 4.38 (0.03–33.90)	79

**Table 4:** Frequency of Patients with Acute Implanted Site-Related Toxicities.

Toxicity	Grade 1 (Mild)		Grade 2 (Moderate)		Grade 3 (Severe)		Total	
	n	%	n	%	n	%	n	%
Dermatitis Radiation	23	32.4%	4	5.6%	0	0%	26	36.6%
Pain at the Implanted Site	9	12.7%	15	21.1%	0	0%	17	23.9%
Pruritis	11	15.5%	0	0%	0	0%	11	15.5%
Localized Edema	3	4.2%	0	0%	0	0%	3	4.2%
Bruising at Implanted Site	3	4.2%	0	0%	0	0%	2	2.8%
Superficial Soft Tissue Fibrosis	1	1.4%	1	1.4%	0	0%	2	2.8%
Wound Infection	0	0%	9	12.7%	0	0%	2	2.8%
Mucositis Oral	1	1.4%	0	0%	0	0%	1	1.4%
Hypertension	0	0%	0	0%	1	1.4%	1	1.4%

## Data Availability

Qualified researchers may request access to the respective study documents (including the clinical study protocols with any amendments and blank case report forms) that support the methods and findings described in this article following the completion of the respective studies. Such researchers shall provide a proposal sufficiently demonstrating scientifically and commercially sound research intentions. Individual anonymized participant data will likewise be made available if there is a legal basis upon which to share the data and there is not a reasonable likelihood of participant re-identification. Requests should be submitted via the corresponding author.
